# Intermittent exercise alleviates MI-induced renal injury in mice via IGF-1

**DOI:** 10.3389/fphys.2025.1733425

**Published:** 2025-12-19

**Authors:** Wanyu Zhu, Wenyan Bo, Yixuan Ma

**Affiliations:** 1 School of Physical Education, Institute of Sports and Exercise Biology, Shaanxi Normal University, Xi’an, Shaanxi, China; 2 College of Physical Education, Shanxi University, Taiyuan, Shanxi, China

**Keywords:** exercise, heart, kidney, IGF-1, PI3K, AKT pathway

## Abstract

Myocardial infarction (MI) often induces acute kidney injury (AKI) via systemic hypoperfusion and oxidative stress, yet the protective mechanisms of exercise remain unclear. This study investigated whether intermittent exercise alleviates MI-induced AKI through the insulin-like growth factor-1 (IGF-1)/PI3K/AKT signaling pathway. An AKI model was established in mice via coronary artery ligation, followed by moderate-intensity intermittent treadmill training for 4 weeks. Echocardiography, serum biochemical markers, renal histology, RT-qPCR, and Western blotting were used to assess cardiac and renal function, inflammatory cytokines, oxidative stress, apoptosis, and IGF-1/PI3K/AKT signaling. *In vitro*, H_2_O_2_-treated NRK renal cells were used to mimic oxidative damage. Recombinant human IGF-1 (rhIGF-1), AMPK agonist AICAR, IGF-1 receptor inhibitor NVP-AEW541, and PI3K inhibitor LY294002 were applied to explore the pathway’s involvement in exercise-induced renoprotection. MI led to impaired cardiac function, renal structural injury, elevated BUN and MDA levels, increased expression of IL-6, TNF-α, Bax, and Cleaved Caspase-3, and decreased SOD activity. Intermittent exercise improved cardiac output, attenuated renal injury, enhanced antioxidant capacity, and upregulated IGF-1 expression and its downstream PI3K/AKT signaling. *In vitro*, rhIGF-1 and AICAR mimicked the protective effects of exercise, while IGF-1R or PI3K inhibitors partially abolished these effects. These findings suggest that intermittent exercise ameliorates MI-induced AKI by activating the IGF-1/PI3K/AKT pathway, thereby exerting anti-inflammatory, antioxidant, and anti-apoptotic effects. This study highlights the role of exercise-induced IGF-1 in heart-kidney axis protection and provides a mechanistic basis for therapeutic interventions targeting MI-related renal complications.

## 1 Introduction

Myocardial infarction (MI), with its increasing prevalence, is a major culprit of cardiac dysfunction and ultimately heart failure (Elgendy et al., 2019). In the acute stage, extensive loss of cardiomyocytes diminishes the heart’s pumping efficiency, leading to reduced cardiac output and systemic hypoperfusion, among which the kidney is particularly susceptible. Clinical observations indicate that a significant proportion of MI patients present with impaired renal function and disturbances in water–electrolyte balance, thereby fostering a detrimental “heart-kidney” interaction, termed cardiorenal syndrome (CRS) (McCallum and Sarnak, 2023; Rangaswami et al., 2019). Therefore, it is of great clinical significance to develop approaches that can concurrently enhance cardiac performance and maintain renal metabolic stability to slow disease progression (Bridgewater et al., 2005).

Current therapeutic strategies for CRS mainly focus on supportive care, including hemodialysis, diuretics, and positive inotropic agents. Although these methods can transiently relieve organ stress, they do not reverse the reciprocal damage between the heart and kidney nor address the underlying pathophysiological mechanisms of the “heart-kidney axis.” In recent years, physical exercise, recognized as a cost-effective and low-risk non-pharmacological intervention, has attracted increasing attention in chronic disease prevention and rehabilitation (Kouidi et al., 2024). Research evidence suggests that exercise, with intermittent exercise in particular, can improve distant organ function by modulating circulating humoral factors and extracellular vesicle content (Chow et al., 2022; Martinez et al., 2021; Gao et al., 2021; Sprick et al., 2022). Among these exercise-induced mediators, insulin-like growth factor-1 (IGF-1) has been identified as a key molecule with diverse biological activities (Fang et al., 2023; Vinciguerra et al., 2012). IGF-1 not only contributes to cardiovascular stability (Lee et al., 2024) but also confers anti-apoptotic, antioxidant, and reparative benefits to renal tissue (Dong et al., 2019). Importantly, levels of IGF-1 in the kidney decrease significantly following MI, whereas exogenous IGF-1 supplementation improves renal function (Cui and He, 2022), highlighting its crucial role in modulating the “heart–kidney axis.”

Exercise has been reported to improve cardiac function after MI partly by upregulating IGF-1 expression (Khetarpal et al., 2025). Previous studies have revealed that IGF-1 exerts cytoprotective effects by binding to its receptor (IGF-1R) and activating the downstream PI3K/AKT signaling pathway (Khan et al., 2025; Meng et al., 2021; Wang et al., 2020; Zeng et al., 2021; Liao et al., 2018; Higashi et al., 2010; Chapuis et al., 2010). Building upon this evidence, previous studies have demonstrated that exercise promotes growth factor–driven recovery of skeletal muscle dysfunction following MI (Li et al., 2022). In particular, exercise activates IGF-1 and its downstream PI3K/AKT signaling cascade, thereby mitigating skeletal muscle atrophy (Feng et al., 2022; Cai et al., 2018). Consistent with these findings, our earlier work revealed that exercise elevates the expression of the myokine Irisin, which subsequently stimulates the AMPK/Sirt1 pathway and suppresses apoptosis in renal cells of MI mice (Wu et al., 2020). Collectively, these results reinforce the notion of an “exercise–myokine–kidney” protective axis. However, whether IGF-1 mediates the renoprotective actions of exercise following MI through the IGF-1R/PI3K/AKT pathway has not been systematically investigated. To address this knowledge gap, we established a murine model of MI-induced acute kidney injury combined with moderate-intensity intermittent treadmill training, and further constructed an *in vitro* oxidative stress model using Normal Rat Kidney (NRK) cells. By applying the IGF-1R inhibitor NVP-AEW541, the PI3K inhibitor LY294002, and human recombinant IGF-1 protein (rhIGF-1). This study aimed to clarify whether exercise-induced activation of the IGF-1/IGF-1R/PI3K/AKT pathway mediates renal protection following MI thereby providing mechanistic insight for developing exercise-based strategies against cardiorenal dysfunction.

## 2 Materials and methods

### 2.1 Experimental animals, cell treatments and grouping

Forty healthy male C57BL/6J mice (8 weeks old, 20–22 g) were purchased from the Animal Experimentation Center of Xi’an Jiaotong University. All procedures conformed to the Guide for the Care and Use of Laboratory Animals (8th ed., ISBN-10: 0-309-15396-4) and were approved by the Animal Ethics Committee of Shaanxi Normal University. Mice were housed at 22 °C–25 °C with 50%–60% humidity under a 12-h light/dark cycle with free access to food and water. After 1 week of acclimation, they were randomly assigned to four groups (n = 10 each): sham (S), sham + exercise (SE), myocardial infarction (MI) and myocardial infarction + exercise (ME).

**FIGURE 1:**
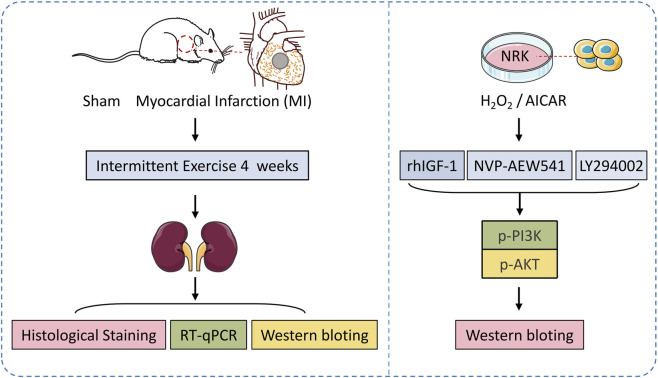
Experimental design and grouping diagram.

Normal rat kidney (NRK) cells were obtained from CytoCell. Cells were maintained in DMEM/F-12 supplemented with 10% fetal bovine serum (Gibco) and 1% penicillin–streptomycin at 37 °C in a humidified 5% CO_2_ incubator. When confluence reached 70%–80%, cells were exposed to 0.2 mmol L^−1^ H_2_O_2_ for 4 h to induce acute oxidative stress, after which they immediately received pharmacological treatments. Interventions included recombinant human IGF-1 (rhIGF-1, PeproTech, 100 ng mL^−1^), the AMPK agonist AICAR (Sigma-Aldrich, 500 µM), the IGF-1R inhibitor NVP-AEW541 (Selleckchem, 5 µM), the PI3K inhibitor LY294002 (Selleckchem, 10 µM), and freshly prepared H_2_O_2_ (0.2 mmol L^−1^ in sterile PBS). All agents were added directly after modelling, and treatment durations (typically 12–24 h) were set according to subsequent protein or gene-expression assays to evaluate their protective effects and signalling impacts following oxidative stress (Figure 1).

### 2.2 Myocardial-infarction model construction and exercise protocol

Myocardial-infarction model construction: After induction of anaesthesia with inhaled isoflurane, each mouse was fixed supine on the surgical table. A mid - sternal thoracotomy was performed to expose the heart, and the left anterior descending coronary artery was ligated. ST-segment elevation or T-wave inversion on the postoperative electrocardiogram, together with visible blanching of the apical myocardium, was taken as evidence of successful modelling

Exercise protocol: One week after surgery, mice underwent adaptive incremental treadmill training _(5–10 m min_^−1^_, 10–30 min d_^−1^_, 5 consecutive days)_. Formal training consisted of a 10-min warm-up at 5 m min^−1^_(≈40–50% VO2max)_, followed by intermittent running: 3 min at 8 m min^−1^_(50–60% VO2max)_ alternated with 7 min at 12 m min^−1^_(80–90% VO2max)_ for a total of 50–60 min d^−1^, 5 days week^−1^ for 4 weeks. The VO_2max_ values were set according to previous measurements and were not determined individually for each animal. Exercise intensity was determined based on VO_2max_ values reported in prior studies using comparable mouse models rather than individual testing, to minimize stress and variability. This method has been widely applied to ensure reproducibility of moderate-intensity protocols (Huang et al., 2025; Mohebinejad et al., 2025; Li et al., 2022).

### 2.3 Cardiac-function assessment

The day after the 4-week training period, mice were anaesthetised with isoflurane, fixed supine and depilated. M-mode echocardiography was performed with a small-animal probe to measure the left-ventricular internal diameter in systole (LVIDs) and diastole (LVIDd), and to calculate ejection fraction (EF) and fractional shortening (FS). For each parameter, the mean of six consecutive cardiac cycles was recorded.

### 2.4 Histopathological examination of kidney

Immediately after the final session, kidneys were rapidly excised, rinsed in ice-cold saline, and partly fixed in 4% paraformaldehyde for 48 h. Samples were dehydrated through a graded ethanol series, cleared in chloroform, embedded in paraffin and sectioned serially at 5 µm. Sections were stained with haematoxylin–eosin (HE; Shanghai Solarbio, G1120) and periodic-acid–Schiff (PAS; Shanghai Solarbio, G1280), dehydrated, cleared with xylene and mounted with neutral resin. Tubular epithelial swelling, cell desquamation, nucleocytoplasmic separation and tubular atrophy were examined and photographed under an Olympus BX51 light microscope, and quantitative analysis was performed with Image-Pro Plus software.

### 2.5 RT-qPCR analysis of renal tissue

Total RNA from renal tissues and cells was extracted with an RNA Rapid Extraction Kit (Beijing Polymed Biosciences). Reverse transcription was carried out using the M5 Super Plus qPCR RT Kit with gDNA Remover in a 20 µL reaction to generate cDNA. Real-time quantitative PCR was performed with 2× M5 HiPer SYBR Premix EsTaq in a 20 µL system under the following programme: 95 °C for 3 min, then 40 cycles of 95 °C for 10 s and 51 °C for 30 s. GAPDH served as the internal control, and relative expression was calculated by the 2^-ΔΔCt method. All qPCR primers were designed by Beijing Qingke Biotechnology; sequences are listed in Table 1.

**TABLE 1:** Sequence list of RT-qPCR primers.

Gene	Forward sequence 5‘–3’	Reverse sequence 5‘–3’
GAPDH	TCACCATCTTCCAGGAGCGAGAC	TGAGCCCTTCCACAATGCCAAAG
AKT	GAGGCGGAAAGAGTGTGTGA	CCAGTGTGAGCCAGAAGTCA
Bax	GTGGAGATGAACTGGACAGCA	GCCACAAAGATGGTCACGGT
Bcl-2	GGTGAACTGGGGGAGGATTG	CGGTTCAGGTACTCAGTCATCC
C-Caspase-3	GGAGTCTGACTGGAAAGCCGAA	CTTCTGGCAAGCCATCTCCTCA
FOXO3a	CAAGAACACCAGCAGCAAAG	TCCTTCCAGCTCCATCTCCT
HO-1	TGCCACCAAGGACCCATAC	TGTGTGCTTGCAATGAGAGTGT
IGF-1	CAGCACTGGGCAGCTCCAT	GGCACTTGCCTCAGAGCACT
IGF-1R	TGGGACAGATCTTGGACTGG	AGTGTCGTCTGCCAAGGTCT
IL-1β	GCAACTGTTCCTGAACTCAACT	ATCTTTTGGGGTCCGTCAACT
IL-6	GAGGATACCACTCCCAACAGACC	AAGTGCATCATCGTTGTTCATACA
KIM-1	TGCCTGGTCTGCACTGTCTA	AGGGTGATGATGGTGACGGA
NF-κB	CGATCAGTACCGGCAGTTGA	GTAGGAGATGGGGTTGGTCTG
Nrf2	GGGATCCCAACTTCCCTGAT	CTGGATCTGGGATGACTGGA
PI3K	ATGGAGAGCCAGTTGGAAAAG	CAGGTTCCCTCAGCTCCATA
SOD1	GGTGTCCGTGTTGTGTTGGT	TCTCGGTGGGTTTCCAGTTA
TNF-α	GGTGCCTATGTCTCAGCCTCTT	GCCATAGAACTGATGAGAGGGAG

### 2.6 Western-blot protein expression analysis

Approximately 50 mg of kidney tissue was homogenised on ice in lysis buffer (RIPA:PMSF: phosphatase inhibitor = 100:1:1). After centrifugation at 12,000 rpm for 15 min, supernatants were quantified by the BCA method, adjusted to 4 ng μL^−1^ and denatured at 100 °C for 10 min. Samples were separated on SDS–PAGE gels and transferred to NC membranes at 300 mA under cooling. Membranes were blocked with 3% BSA for 2 h, then probed with primary antibodies against IGF-1 (Abcam, ab133542), IGF-1R (Abcam, ab182408), PI3K (CST, 4257S), AKT (CST, 9272S), TNF-α (CST, 11948S), IL-6 (Abcam, ab9324), Cleaved Caspase-3 (CST, 9661) and GAPDH (Abcam, ab181602). After 2 h at room temperature, membranes were incubated with HRP-conjugated secondary antibodies, washed five times with TBST and developed with ECL; band intensities were quantified using a multicolour imaging system.

### 2.7 Kit-based biochemical assays

For biochemical assays, reagents were added sequentially to 96-well plates, and absorbance was measured on a microplate reader at kit-specified wavelengths: SOD (Nanjing Jiancheng, A001-3, 550 nm), MDA (Nanjing Jiancheng, A003-1, 532 nm) and BUN (Nanjing Jiancheng, C013-2, 520 nm) were measured in kidney homogenates, while IGF-1 (R&D Systems, MIG100, 450 nm) was determined in serum.

### 2.8 Data processing and statistical analysis

Images were processed with ImageJ, Western blot data with Image Lab, and statistical analysis with GraphPad Prism 9. One-way ANOVA or linear regression was applied; multiple comparisons used Tukey’s test. Data are presented as mean ± SD. *p < 0.05 indicated statistical significance, **p < 0.01 high significance and ***p < 0.001 very high significance.

## 3 Results

### 3.1 Cardiac-function results in mice

**FIGURE 2:**
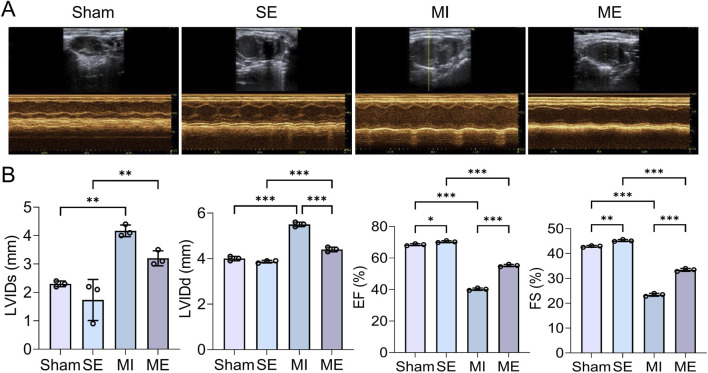
Evaluation of cardiac function in MI mice subjected to intermittent exercise. **(A,B)** Representative echocardiographic images and quantitative measurements of cardiac parameters. Data are presented as mean ± SD. n = 4 mice per group. Each experiment was repeated at least four times independently. *p < 0.05, **p < 0.01, ***p < 0.001. LVIDd, left ventricular internal diameter during diastole; LVIDs, left ventricular internal diameter during systole; EF, ejection fraction; FS, fractional shortening. Groups: Sham-sham-operated; SE, sham + exercise; MI, myocardial infarction; ME, myocardial infarction + exercise.

Myocardial echocardiography showed that ventricular systolic motion was weakened in the MI group compared with the S group, whereas the ME group displayed an improvement (Figure 2A). LVIDs (3.21 ± 0.15 mm) and LVIDd (4.58 ± 0.12 mm) were significantly increased, while EF (42.3% ± 3.5%) and FS (21.8% ± 2.1%) decreased compared with S mice (EF 68.5% ± 2.9%, FS 36.2% ± 1.8%; *p* < 0.001). In contrast, the ME group exhibited smaller LVIDs and LVIDd and higher EF and FS than the MI group (*p* < 0.001) (Figure 2B). These findings confirm successful construction of the MI model—cardiac function declined in MI mice—while intermittent exercise safely and effectively improved cardiac performance in the ME group.

### 3.2 Intermittent exercise improves MI-induced renal injury in mice

**FIGURE 3:**
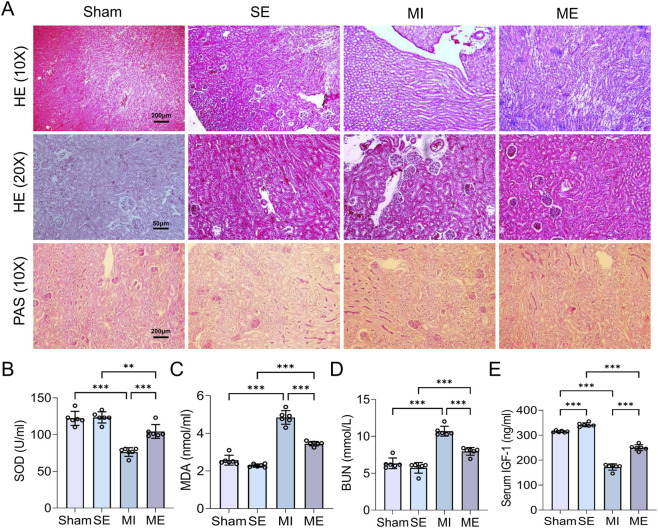
Histological and biochemical evaluation of renal injury following MI and intermittent exercise. **(A)** Hematoxylin–eosin (HE; magnifications 10×, 20×) and periodic acid–Schiff (PAS) staining of kidney sections. **(B–E)** Quantitative determination of SOD activity, MDA levels, BUN concentration, and IGF-1 content. Data are presented as mean ± SD. n = 4 mice per group. Each experiment was repeated at least six times independently. *p < 0.05, **p < 0.01, ***p < 0.001. SOD, superoxide dismutase; MDA, malondialdehyde; BUN, blood urea nitrogen; IGF-1, insulin-like growth factor-1.

Histopathology of the kidney revealed intact architecture with no evident injury in S and SE mice on both HE and PAS staining. In MI mice, tubular epithelial swelling and detachment, interstitial oedema and tissue damage were observed; In ME mice, pathological changes were attenuated, with relatively preserved tubular structure and less interstitial damage (Figure 3A). Biochemically, MI mice displayed significantly higher serum BUN and MDA levels and lower SOD activity and IGF-1 concentrations than controls (*p* < 0.001). Intermittent exercise reduced BUN and MDA while elevating SOD and IGF-1, all with high statistical significance (*p* < 0.001) (Figures 3B–E). Thus, MI induces renal injury and dysfunction, whereas intermittent exercise substantially alleviates kidney damage and restores renal function.

### 3.3 Intermittent exercise upregulates renal IGF-1 and its pathway in MI mice and modulates genes for inflammation, apoptosis and oxidative stress

**FIGURE 4:**
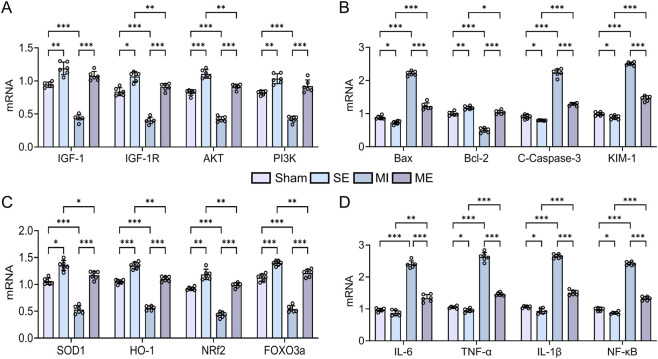
Relative mRNA expression of IGF-1 pathway components and related molecular markers in renal tissue. **(A)** IGF-1 and downstream signaling genes. **(B)** Apoptosis-related genes. **(C)** Oxidative stress markers. **(D)** Inflammatory cytokines. Data are presented as mean ± SD. n = 4 mice per group. Each experiment was repeated at least six times independently. *p < 0.05, **p < 0.01, ***p < 0.001.

Pro-apoptotic genes, pro-inflammatory genes and the kidney-injury marker KIM-1 were significantly upregulated in the MI group versus the S group (*p* < 0.01 or *p* < 0.001), whereas the anti-apoptotic gene Bcl-2, antioxidant genes and genes in the IGF-1/IGF-1R/PI3K/AKT pathway were downregulated (*p* < 0.01 or *p* < 0.001). Compared with the MI group, the ME group showed reductions in pro-apoptotic, pro-inflammatory and injury-related transcripts (*p* < 0.01 or *p* < 0.001) and significant increases in anti-apoptotic, antioxidant and IGF-1-pathway transcripts (*p* < 0.01 or *p* < 0.001) (Figures 4A–D). These results indicate that MI suppresses renal IGF-1 expression and its downstream anti-inflammatory, anti-apoptotic and anti-oxidative signalling, whereas intermittent exercise re-activates the IGF-1 axis and mitigates MI-induced renal injury, thereby conferring renoprotection.

### 3.4 Intermittent exercise enhances renal IGF-1 signalling and regulates inflammatory proteins in MI mice

**FIGURE 5:**
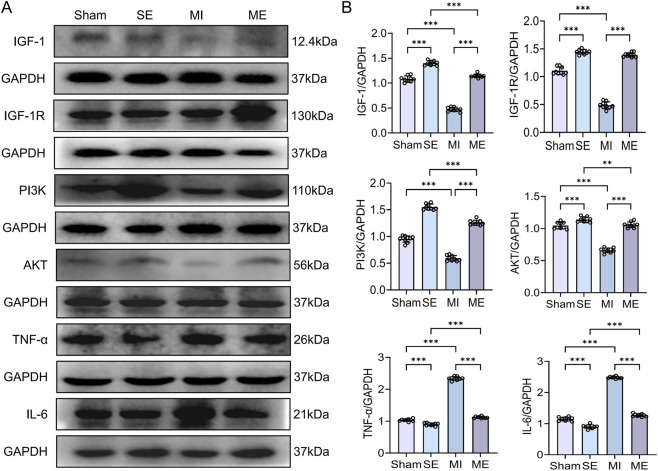
Protein expression of IGF-1/PI3K/AKT signaling components and inflammatory cytokines in renal tissue. **(A,B)** Immunoblotting results for IGF-1, IGF-1R, PI3K, AKT, TNF-α, and IL-6. Data are presented as mean ± SD. n = 4 mice per group. Each experiment was repeated nine times independently. *p < 0.05, **p < 0.01, ***p < 0.001. IGF-1R, insulin-like growth factor-1 receptor; TNF-α, tumor necrosis factor-alpha; IL-6, interleukin-6.

Protein analysis showed that IGF-1, IGF-1R, PI3K and AKT were reduced in MI mice compared with S mice (*p* < 0.001), whereas the inflammatory proteins TNF-α and IL-6 were significantly elevated (*p* < 0.001). In ME mice, IGF-1, IGF-1R, PI3K and AKT protein levels were significantly higher, and TNF-α and IL-6 lower, than in MI mice (*p* < 0.001) (Figures 5A,B). These findings confirm that MI depresses the renal IGF-1 pathway and its anti-inflammatory capacity, while intermittent exercise re-activates this pathway and alleviates MI-induced kidney damage. Western blot analysis was performed for both total and phosphorylated forms of pathway proteins where available. Total protein levels were used as an indirect indicator of signaling activation, consistent with previous studies reporting parallel changes in phosphorylation status.

### 3.5 Exogenous AICAR and rhIGF-1 activate PI3K/AKT and suppress H_2_O_2_-induced cellular inflammation

**FIGURE 6:**
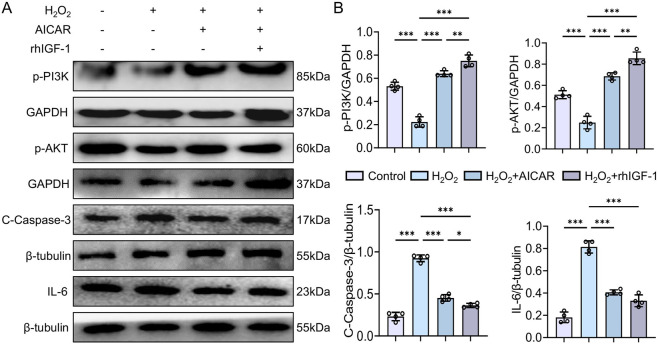
Effects of ALCAR and rhIGF-1 on PI3K/AKT pathway activation and inflammation in H_2_O_2_-treated NRK cells. **(A,B)** Protein levels of p-PI3K, p-AKT, Cleaved Caspase-3, and IL-6 after AICAR ± rhIGF-1 treatment. Data are presented as mean ± SD. n = 4 wells per group. Each experiment was repeated at least four times independently. * *p* < 0.05, ** *p* < 0.01, *** *p* < 0.001.

Compared with the control group (C), the H_2_O_2_ group showed a marked reduction in p-PI3K and p-AKT expression, accompanied by significant elevations in the inflammatory factors IL-6 and Cleaved Caspase-3 (*p* < 0.01 or *p* < 0.001). Both the H_2_O_2_ + AICAR and H_2_O_2_ + rhIGF-1 treatments significantly increased p-PI3K and p-AKT levels while lowering IL-6 and Cleaved Caspase-3 (*p* < 0.01 or *p* < 0.001) (Figure 6). These findings indicate that H_2_O_2_ exposure in NRK cells mimics MI-related renal injury, whereas exogenous rhIGF-1 or AICAR enhances IGF-1 signalling and activates the downstream PI3K/AKT pathway, thereby attenuating H_2_O_2_-induced inflammation and cellular damage.

### 3.6 Exogenous NVP-AEW541 inhibits PI3K/AKT and exacerbates H_2_O_2_-induced cellular inflammation

**FIGURE 7:**
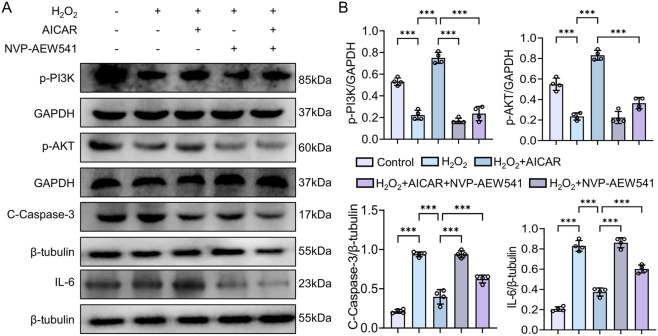
Influence of IGF-1R inhibition on AICAR-mediated effects in H_2_O_2_-treated NRK cells. **(A,B)** p-PI3K, p-AKT, Cleaved Caspase-3, and IL-6 expression after AICAR ± NVP-AEW541 treatment. Data are presented as mean ± SD. n = 4 wells per group. Each experiment was repeated at least four times independently. * *p* < 0.05, ** *p* < 0.01, *** *p* < 0.001.

In the H_2_O_2_ + AICAR group, p-PI3K and p-AKT were strongly upregulated, whereas IL-6 and Cleaved Caspase-3 were suppressed (*p* < 0.01 or *p* < 0.001). When the IGF-1R inhibitor NVP-AEW541 was added (H_2_O_2_ + NVP-AEW541), p-PI3K and p-AKT levels fell sharply and IL-6 and Cleaved Caspase-3 rebounded (*p* < 0.01 or *p* < 0.001) (Figure 7). Thus, the cytoprotective effect of AICAR, which mimics exercise, depends largely on IGF-1R-mediated activation of the PI3K/AKT pathway to exert anti-inflammatory actions.

### 3.7 Exogenous LY294002 blocks PI3K/AKT and worsens H_2_O_2_-induced cellular inflammation

**FIGURE 8:**
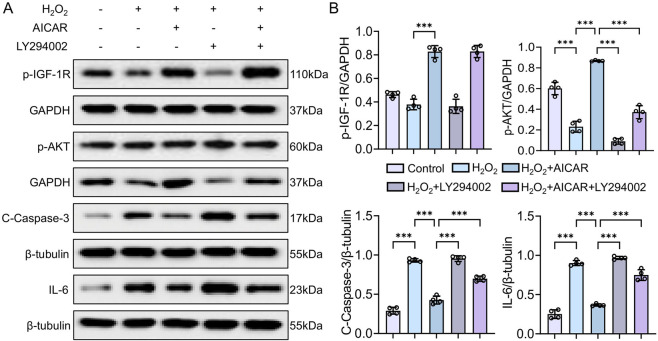
Effect of PI3K inhibition on AICAR-induced signaling changes in H_2_O_2_-treated NRK cells. **(A,B)** p-IGF-1R, p-AKT, Cleaved Caspase-3, and IL-6 protein expression following AICAR ± LY294002 treatment. Data are presented as mean ± SD. n = 4 wells per group. Each experiment was repeated at least four times independently. * *p* < 0.05, ** *p* < 0.01, *** *p*< 0.001.

The H_2_O_2_ group suppressed p-IGF-1R and p-AKT while elevating IL-6 and Cleaved Caspase-3 (*p* < 0.01 or *p* < 0.001). In contrast, the H_2_O_2_ + AICAR group significantly increased p-IGF-1R and p-AKT and reduced IL-6 and Cleaved Caspase-3 (*p* < 0.01 or *p* < 0.001). When PI3K was blocked with LY294002 (H_2_O_2_ + LY294002), activation of p-AKT was impeded, IL-6 and Cleaved Caspase-3 rebounded, and p-IGF-1R was likewise suppressed (*p* < 0.01 or *p* < 0.001) (Figure 8). These results demonstrate that the PI3K/AKT pathway is indispensable for IGF-1-mediated anti-inflammatory protection in renal cells.

## 4 Discussion

The kidney plays a central role in metabolism and waste removal, and its health is vital to the maintenance of overall physiological stability. Clinical and experimental findings have shown that ischemia/reperfusion injury, oxidative stress, and inflammation can rapidly induce both structural and functional damage to renal tissue. MI often leads to secondary kidney impairment, presenting either as acute cardiogenic injury caused by hypoperfusion or as chronic congestive nephropathy resulting from persistent hypoxia; these conditions are collectively recognized as CRS (Rangaswami et al., 2019; Young and Eknoyan, 2024; Zannad and Rossignol, 2018). In clinical settings, this often manifests as edema, which elevates cardiac preload and sustains the detrimental cycle of heart–kidney dysfunction (Schefold et al., 2016).

In this study, we employed a mouse model of acute MI combined with a program of intermittent exercise to evaluate whether physical training could counteract MI-induced kidney injury. We further investigated potential mechanisms by assessing the activation status of the renal IGF-1/IGF-1R/PI3K/AKT pathway, alongside markers of oxidative stress, inflammation, and apoptosis.

After MI, reduced cardiac output leads to inadequate systemic perfusion, causing structural and functional injury in peripheral organs, with the kidney particularly affected. Contributing factors include reduced renal blood flow, heightened sympathetic activation, and excessive stimulation of the RAAS, all of which promote tubular hypoxia and cellular damage, ultimately resulting in cardiogenic acute kidney injury (Young and Eknoyan, 2024; Zannad and Rossignol, 2018). In our model, MI mice exhibited significant cardiac dysfunction and biochemical signs of renal oxidative stress. Four weeks of intermittent exercise training reversed these changes, improving EF and FS, restoring ventricular dimensions, reducing BUN levels, increasing SOD activity, and lowering MDA concentrations. These results are consistent with previous findings demonstrating that exercise regulates oxidative balance, thereby exerting protective effects on renal function in diverse pathological conditions (Watson et al., 2022).

Renal tubular epithelial cells, essential for glomerular filtration and solute reabsorption, are highly sensitive to oxidative stress, inflammation, and apoptosis during MI-induced injury (Zhao et al., 2021). IGF-1, a multifunctional myokine secreted by numerous tissues, plays a key role in cellular protection. In the kidney, IGF-1 binding to its receptor (IGF-1R) activates the PI3K/AKT pathway, thereby counteracting oxidative damage and suppressing apoptotic signaling (Kamenický et al., 2014; Khan et al., 2025). Exercise combined with IGF-1 has been shown to reduce Sirt1 expression, thereby alleviating cellular senescence and attenuating myocardial injury (Meng et al., 2021). In our study, MI reduced IGF-1 expression, downregulated PI3K/AKT/FOXO3a activity, and diminished Sirt1 levels, while simultaneously elevating markers of oxidative stress, pro-inflammatory cytokines, and apoptotic proteins. Intermittent exercise restored IGF-1 and IGF-1R expression, enhanced PI3K/AKT signaling, increased FOXO3a phosphorylation, upregulated Sirt1 expression, and attenuated oxidative, inflammatory, and apoptotic responses, which is consistent with previous findings that exercise-induced IGF-1 upregulation mitigates multi-organ injury (Cui et al., 2020; Ibañez et al., 2025).

The IGF-1/IGF-1R/PI3K/AKT pathway is widely recognized as a major regulator of cell growth, differentiation, antioxidant defense, and anti-apoptotic responses, making it a key mediator in tissue repair (Ye et al., 2024; Cui and He, 2022). In this work, intermittent exercise not only reactivated this signaling cascade but also reduced inflammatory mediators, oxidative stress, and apoptotic markers, while lowering KIM-1 expression, indicating less renal injury. This study used group-based VO_2max_ reference values to determine exercise intensity, which may not reflect individual physiological variation. In addition, total protein levels were used as proxies for pathway activation due to limited tissue availability, and future studies should incorporate phosphorylated protein assessment for confirmation.

Overall, our findings demonstrate that intermittent exercise alleviates MI-associated kidney damage through IGF-1/IGF-1R/PI3K/AKT pathway activation and the suppression of pathological oxidative, inflammatory, and apoptotic processes. These results provide mechanistic insight into the benefits of exercise for preserving kidney function after MI and support the translation of targeted exercise prescriptions into clinical practice for patients at risk of cardiorenal syndrome. Despite these promising findings, limitations include the use of total rather than phosphorylated protein analysis, the absence of individual VO_2max_ testing, and lack of long-term follow-up. Future studies should address these issues to strengthen translational relevance.

## 5 Conclusion

**FIGURE 9:**
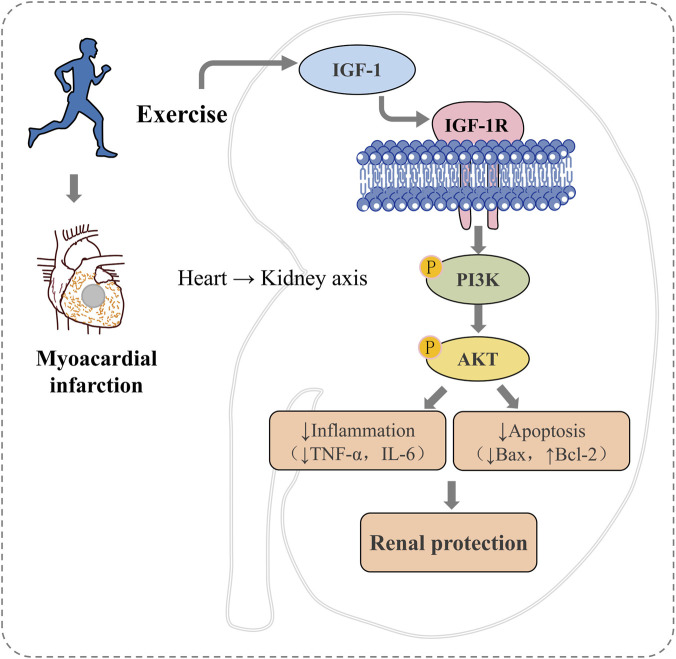
Proposed mechanistic model.

Myocardial infarction triggers renal oxidative stress, inflammation and apoptosis, culminating in functional impairment. Intermittent exercise attenuates these pathological changes, an effect closely associated with upregulation of renal IGF-1 and activation of the IGF-1R/PI3K/AKT signalling axis. Activation of this pathway suppresses pro-inflammatory cytokine release, lowers oxidative-stress levels and inhibits apoptosis, thereby preserving renal structure and function (Figure 9). These findings identify IGF-1 as a pivotal molecular target through which intermittent exercise mitigates MI-induced secondary kidney injury, offering new theoretical insight and potential therapeutic avenues for the management of cardiorenal syndrome.

## Data Availability

The original contributions presented in the study are included in the article/supplementary material, further inquiries can be directed to the corresponding author.
